# Lower In-Hospital Mortality with Plasma Exchange than Plasmapheresis in a Subgroup Analysis of 374 Lupus Patients

**DOI:** 10.1155/2018/9707932

**Published:** 2018-05-02

**Authors:** Yu-Jih Su, Wen-Chan Chiu, Chung-Yuan Hsu, Jin-Bor Chen, Hwee-Yeong Ng

**Affiliations:** ^1^Division of Rheumatology, Allergy and Immunology, Department of Internal Medicine, Kaohsiung Chang Gung Memorial Hospital and Chang Gung University College of Medicine, Kaohsiung, Taiwan; ^2^Division of Nephrology, Department of Internal Medicine, Kaohsiung Chang Gung Memorial Hospital and Chang Gung University College of Medicine, Kaohsiung, Taiwan

## Abstract

**Background:**

Apheresis treatment includes plasmapheresis (PP) and plasma exchange (PE), and these terms are commonly used interchangeably. Nevertheless, the two procedures are carried out differently. The aims of this study were to investigate the mortality rate of patients who underwent therapeutic apheresis and compare the mortality rate between PP and PE.

**Methods:**

We conducted a medical chart review retrospective study. All identified subjects (*n* = 436) were over 20 years old with at least one ICD-9-CM intervention code plasmapheresis or plasma exchange and at least one diagnosis code with rheumatic disease. All of them were hospitalized to Chang Gung Memorial Hospital between 1st of January, 2000, and 31st of December, 2014.

**Results:**

436 nonoverlapping patients had never received PE and/or PP before 1 Jan, 2000. Among all the patients, 350 received PE, 63 received PP, and 23 received both therapies. Female patients accounted for 85.09% of patients. The overall mortality rate was 4.65% in the PE subgroup, 4.76% with combination therapy, and 13.46% in the PP subgroup. There were 374 patients diagnosed as SLE, which is the majority of overall patients who received PE and/or PE. In multivariate analysis, PE was the sole independent factor predictor of survival in SLE subgroup patients (*p* = 0.02, exp(*B*) = 0.314, 95% CI 0.12–0.81).

**Conclusions:**

We showed that both PP and PE were used in treating a variety of autoimmune disorders. Plasmapheresis was preferentially carried out in patients with peripheral neuropathy. In 374 lupus patients treated with either PE or PP, PE is superior to PP in reducing in-hospital mortality.

## 1. Introduction

Systemic lupus erythematosus (SLE) is an autoimmune disease, which is characterized by the presence of a wide profile of autoantibodies. Several autoantibodies produced by the uninhibited B cells were noticeably associated with specific manifestations [1]. Despite recent advances in both the understanding and treatment of the disease, considerable mortality persists. These patients died more than 3 times more frequently than age- and sex-matched controls from the normal population [2].

Aside from corticosteroid and immunosuppressive agents, therapeutic apheresis has been reported to be effective in treating a variety of serious lupus complications, such as diffuse alveolar hemorrhage [2], catastrophic antiphospholipid syndrome [3], and cerebritis [4]. Apheresis treatment includes double filtration plasmapheresis (PP) and plasma exchange (PE), and these terms are commonly used interchangeably [5]. Nevertheless, the two procedures are carried out differently. Unlike PP, PE requires fluid replacement to maintain homeostasis after bulk removal of plasma [6]. By using the replacement fluid such as human serum albumin and fresh frozen plasma [6], PE may be associated with the possible risks of allergic reactions and transfusion-related infections that are not encountered in PP. Although both procedures are effective in removing large pathogenic substances from the plasma [7], patient outcomes have been inadequately investigated.

The aims of this study were to investigate the mortality rate of lupus patients who underwent therapeutic apheresis and compare the mortality rate between PP and PE. We also aimed to identify the factors that were associated with mortality.

## 2. Materials and Methods

### 2.1. Participants

For this retrospective study, we conducted a medical chart review and identified all subjects (*n* = 436) who were over 20 years old, with at least one ICD-9-CM intervention code (9971 or 9904) and at least one diagnosis code (between 710.0 and 710.9 [rheumatic diseases]), based on one or more admissions to the Chang Gung Memorial Hospital, Kaohsiung (CGMH-KS) between 1st of January, 2000, and 31st of December, 2014. The types of therapeutic apheresis were confirmed by reviewing the medical records manually. The diagnostic code between 710.0 and 710.9 includes systemic lupus erythematosus (SLE), systemic sclerosis, Sjogren's syndrome, dermatomyositis, polymyositis, eosinophilia myalgia syndrome, and other specified and unspecified diffuse connective tissue disease. The 374 patients diagnosed with SLE were selected for detailed chart review and laboratory data analysis. All patients were followed by rheumatologists from CGMH-KS. The CGMH-KS is a tertiary care referral center located in Kaohsiung County (southern Taiwan) that serves a population of about 2 million people.

This is a retrospective study, and informed consent was not obtained from individual patients. All the patient records and information were anonymized and deidentified prior to analysis. This study was conducted according to a protocol approved by the Ethics Committee of the CGMH (IRB number: 102-4669B).

### 2.2. Comparison of Subgroups

For comparisons, patients were divided into three subgroups according to their treatment protocols (PE, PP, or sequential treatments of both, here we refer to it as combination treatment). Some patients received PE and PP as a row, which could be the clinical situation which deteriorated after one treatment or the therapy was not effective with initial therapy; then the clinician switched the therapy to the other modality according to clinical judgment. In this situation, both therapies contribute to the final outcome, life or death of the patient, so that we took both PE and PP into consideration in chi square statistics. Associated diseases were listed and compared between the three subgroups. Furthermore, SLE patients were selected, and laboratory data were collected for analysis. SLE patients were similarly divided into three subgroups according to their treatment protocols, and comparisons were made between these three subgroups.

### 2.3. Data Collection

Demographic and clinical characteristics were collected for all 436 patients including age, gender, leukocyte differential count, hemoglobin, hematocrit, platelet count, inflammation markers (C-reactive protein, erythrocyte sediment rate, and rheumatoid factor), lipid profile, hepatitis B surface antigen, anti-hepatitis C serological marker, cryoglobulin, anti-nuclear antibodies, biochemical laboratory data, and the autoantibodies. The autoantibodies included the anti-extractable nuclear antibodies (anti-ENA) and antiphospholipid antibodies. The anti-ENA antibodies included the anti-Ro, anti-La, anti-Smith, anti-U1 RNP, anti-Scl70, and anti-Jo1 subtypes. The antiphospholipid antibodies included the anti-beta 2 glycoprotein I, anticardiolipin IgG, and anticardiolipin IgM subtypes.

Plasmapheresis and PE were carried out using the HF-440 (Infomed, Geneva, Switzerland). Plasmacure™ PE (Kawasumi Laboratories, Tokyo, Japan), a hollow fiber, was used as the plasma separator to isolate plasma from blood cells and platelets. All patients received PP or PE as an adjuvant therapy when the disorders did not respond to immunosuppressants. The blood flow rate was 100 mL/min and the processed volume was 1.4 times plasma volume (estimated plasma volume (L) = 0.07 × body weight (kg) × (1 − hematocrit)) for each session of apheresis. The isolated plasma, which contains pathogenic proteins or components, will be treated differently according to the apheresis method. In PE, the isolated plasma was replaced by fresh frozen plasma. As for PP, the separated plasma was pumped through the Evaflux 4A (Kawasumi Laboratories, Tokyo, Japan) plasma fractionators to remove the pathogenic molecules. Heparin was used for anticoagulation. (Supplement Table 1).

### 2.4. Statistical Methods

Patient characteristics are given as simple descriptive statistics. The means and standard deviations (SDs) were used to summarize continuous variables, and percentages for data with nonnormal distributions. For continuous variables with nonnormal distribution, arcsine transformation was used to transform the variates into a normal distribution before analysis. In the univariate analysis, categorical and continuous variables were compared using the Fisher's exact test or chi square test and the Student *t*-test, respectively. In the multivariate analysis, a one-way ANOVA was used to compare between the different subgroups. Statistical significance was defined as a *p* value less than 0.05. All analyses were performed using the SPSS software program, version 15.5 (SPSS, Chicago, IL).

## 3. Results

### 3.1. Demographic Data of Participants and Diseases Receiving PE and/or PP

Four hundred thirty-six nonoverlapping patients had never received PE and/or PP in the last 15 years. Among all the patients, 350 received PE, 63 received PP, and 23 received both therapies. Female patients accounted for 85.09% of patients. The diagnosed autoimmune diseases consisted of SLE (85.78%), Sjogren's syndrome (8.49%), systemic sclerosis (3.67%), rheumatoid arthritis (2.06%), dermatomyositis (2.06%), and polymyositis (1.83%). The associated symptoms or diseases included malignancy (3.67%), diabetes mellitus (2.75%), hypertension (8.26%), peripheral neuropathy (1.61%), and non B/non C hepatitis (2.75%) (Table 1).

There were no differences in the preference of apheresis modality for each disease except for peripheral neuropathy (*p* < 0.01). Six out of the 7 patients with peripheral neuropathy were treated with PP, and the other patient received PE and PP during admission. For diagnostic purposes, 22 (5.05%) and 19 (4.36%) out of all 436 patients underwent a kidney and bone marrow biopsy, respectively, during same course of admission.

Among the patients who only received PE (*n* = 350), 86%, 7.14%, 4%, 2.29%, 2.57%, and 1.71% of patients had SLE, Sjogren's syndrome, systemic sclerosis, rheumatoid arthritis, dermatomyositis, and polymyositis, respectively. Among the patients who only received PP (*n* = 63), 82.54%, 14.29%, 3.17%, 1.59%, and 3.17% of patients had SLE, Sjogren's syndrome, systemic sclerosis, rheumatoid arthritis, and polymyositis, respectively (Table 1). The overall mean mortality rate was 5.28%. Lower mortality rates were observed in the PE compared to the PP group (4.29% versus 11.11%) but these did not reach statistical significance (*p* = 0.08).

### 3.2. Yearly Mortality Rate of Lupus Patients Who Received either PE and/or PP

The SLE mortality rate was recorded year by year from 2000 to 2014. The overall mortality rate was 5.88% among SLE patients who received PE or PP. The mortality rate after receiving therapy was highest in 2003, which was 17.39%, and was zero in 2004, 2005, 2006, and 2008. The comparison between each group was listed in Table 2. Statistically significant differences between groups were only reached in 2009, 2010, and the sum of the results from the past 14 years (*p* = 0.03, *p* < 0.01, and *p* = 0.04, respectively.) The overall mortality rate was 4.65% in the PE subgroup, 4.76% with combination therapy, and 13.46% in the PP subgroup (Table 2).

### 3.3. Comparison of Laboratory Data between the Treatment Subgroups

The clinical laboratory data and the titers of autoantibodies of all the 374 SLE patients were recorded by chart review (Table 3). Most of the hemograms were similar between the three subgroups, except the hemoglobin and hematocrit values (*p* = 0.02 and 0.04, respectively). Post hoc analysis showed that both hemoglobin and hematocrit were significantly higher in the patients from the PP group compared to the PE subgroup (*p* = 0.02 and *p* = 0.02, respectively). The age of patients was significantly older in the PE group compared to the combination therapy group (*p* = 0.01). The titers of the C-reactive protein autoantibodies and the erythrocyte sediment rate were similar among the three subgroups (all *p* > 0.05) (Table 3).

### 3.4. Factors Associated with In-Hospital Survival in Lupus Patients with Serious Complications

In order to investigate the possible link between disease pathogenesis and the choices of therapies, we compared the clinical parameters of the survivors and mortalities during hospitalization. In the univariate study, the presence of other comorbidities with SLE was not a risk factor for in-hospital mortality (all *p* > 0.05). Diabetes mellitus seemed to be a risk factor for mortality (*p* = 0.06). Bone marrow and kidney biopsy examinations were not related to mortalities (both *p* > 0.05). The treatment modalities PE (*p* = 0.02) and PP (*p* = 0.04) were associated with the increased chances of survival. In multivariate analysis, PE was the sole independent factor predictor of survival in SLE subgroup patients (*p* = 0.02, exp(*B*) = 0.314, 95% confidence interval 0.12–0.81) (Table 4).

## 4. Discussion

This article illustrates the clinical picture of patients with rheumatic diseases, who received either PE or PP in a medical center in Taiwan. The choice of treatment method depended primarily on the physicians' clinical judgment. In our study, we showed that the mortality rate of lupus patients who received apheresis therapy for serious complications was 5.88%. Significant lower mortality rate was observed in lupus patients who underwent PE, even in the absence of differences in inflammatory and immune markers with the PP group. In the multivariate study, PE (but not PP) was identified as an independent predictor of survival in these critically ill patients.

A few articles discussed the use of these two treatment modalities [8]. Our study showed that peripheral neuropathy is the only disease that was preferentially treated with plasmapheresis (*p* < 0.01, Table 1). Neuropathy has been treated by PP in various conditions including cryoglobulinemia [9], Sjogren's syndrome [10], viral neuropathy [11], and mixed connective tissue disease [12]. Partial responses in patients who had received both PP and PE were shown to be of limited value in patients with multifocal motor neuropathy [13]. Recently, the American Society for Apheresis has updated the indications for therapeutic apheresis. The guidelines showed that PE/PP therapy may be effective in more than 70 kinds of disease, suggesting that PP or PE is another treatment option especially at medical failure [14].

In our study, we found that patients received PE had significantly lower hemoglobin level than patients with PP. Lupus induced anemia can be caused by immunologic or nonimmunologic etiologies. Immunologic etiology is a result of production of autoantibodies and excessive cytokines. Overproduction of inflammatory cytokines induced upregulation of hepcidin [15] and in turn inhibits intestinal iron absorption and iron bioavailability [16]. Decreased production of erythropoietin [17] and inhibition of erythropoiesis progenitor cells by cytokines [18] have also been reported. In addition, increased red blood cell destruction is noted in lupus complications, such as thrombotic microangiopathic hemolytic anemia and autoimmune hemolytic anemia [19]. Anemia reflects the disease severity of lupus [20] and is closely linked to frequent hospitalization [21]. Even baseline hemoglobin was lower in patients treated with PE; their in-hospital mortality was still significantly lower. In multivariate analysis, hemoglobin was not a significant parameter to predict outcome. Taken together, our study suggests that PE may modulate lupus related anemia.

It has been reported that the mortality rates and long term outcomes of patients with Guillain-Barre's syndrome were not markedly different between the PP and PE treatment groups [22]. However, the effect of different therapeutic apheresis on mortality in lupus patients with catastrophic complications remains unknown. In our study, we showed that patients treated with PE had significantly lower mortality rates compared to those treated with PP and PE remained the independent factor affecting lupus mortality in multivariate analysis. Although both treatment modalities have been reported to be beneficial in treating lupus complications, the ability of PP to remove pathogenic immunoglobulins is lower than PE [23, 24]. The efficacy of pathogenic molecules removal of PP is determined or limited by the pore size of plasma fractionator. During acute stage of these autoimmune disorders, replacement with normal plasma in PE may remove more extensive inflammatory mediators and disrupt the vicious cycle driving to inflammation storm. In this setting, PE may provide immediate clearance of pathogenic substances that is crucial to their survival. More studies are needed to confirm this hypothesis.

Since this is a retrospective cohort study, there are several disadvantages and limitations. First, some data were missing and we did not perform a longitudinal follow-up of the patients to ascertain outcomes. Second, there could have been selection bias among the different rheumatic diseases, because the use of the ICD-9 diagnostic codes was at the discretion of the individual physicians. Nevertheless, our result provides some evidence that the mortality rate is significantly higher in SLE patients who received PP than those who received plasma exchange. We still need a larger prospective study to confirm our findings before making any conclusions.

In conclusion, we showed that both PP and PE were used in treating a variety of autoimmune disorders. Plasmapheresis was preferentially carried out in patients with peripheral neuropathy. In lupus patients with catastrophic complications, PE is superior to PP in reducing in-hospital mortality.

## Figures and Tables

**Table 1:** Comparison of patient characteristics according to treatment modality.

*N* = 436			Plasma exchange (*α*)		Combination (*β*)		Plasmapheresis (*γ*)		*p* value
			*n* = 350		*n* = 23		*n* = 63		
Female	371	(85.09%)	299	85.43%	21	91.30%	51	80.95%	0.45
Autoimmune/rheumatic diseases									
Systemic lupus erythematosus	374	(85.78%)	301	86.00%	21	91.30%	52	82.54%	0.57
Sjogren's syndrome	37	(8.49%)	25	7.14%	3	13.04%	9	14.29%	0.13
Systemic sclerosis	16	(3.67%)	14	4.00%	0	0.00%	2	3.17%	0.59
Rheumatoid arthritis	9	(2.06%)	8	2.29%	0	0.00%	1	1.59%	0.73
Dermatomyositis	9	(2.06%)	9	2.57%	0	0.00%	0	0.00%	0.32
Polymyositis	8	(1.83%)	6	1.71%	0	0.00%	2	3.17%	0.58
Rheumatism (others)	5	(1.15%)	4	1.14%	0	0.00%	1	1.59%	0.83
Other comorbidities									
Malignancy	16	(3.67%)	16	4.57%	0	0.00%	0	0.00%	0.13
Diabetes mellitus	12	(2.75%)	12	3.43%	0	0.00%	0	0.00%	0.22
Hypertension	36	(8.26%)	32	9.14%	3	13.04%	1	1.59%	0.09
Hepatitis without HBV or HCV	12	(2.75%)	11	3.14%	0	0.00%	1	1.59%	0.56
Cytomegalovirus infection	2	(0.46%)	1	0.29%	0	0.00%	1	1.59%	0.35
Chronic hepatitis B infection	1	(0.23%)	1	0.29%	0	0.00%	0	0.00%	0.88
Hepatitis C infection	1	(0.23%)	1	0.29%	0	0.00%	0	0.00%	0.88
Peripheral neuropathy	7	(1.61%)	0	0.00%	1	4.35%	6	9.52%	<0.01^*∗*^
Gout	1	(0.23%)	1	0.29%	0	0.00%	0	0.00%	0.88
Hyperlipidemia	4	(0.92%)	3	0.86%	1	4.35%	0	0.00%	0.17
Major depression	2	(0.46%)	1	0.29%	0	0.00%	1	1.59%	0.35
Fibromyalgia	4	(0.92%)	3	0.86%	0	0.00%	1	1.59%	0.76

*∗* indicates *p* < 0.05; the comparison is significant between *α* and *γ*, as well as between *α*, *β*, and *γ*.

**Table 2:** Comparison of survival and mortality rates between systemic lupus erythematosus patients according to treatment modality.

Treated systemic lupus erythematosus cases *n* = 374		Discharge condition		Subtotal	Plasma exchange (*α*)			Plasma exchange & plasmapheresis (*β*)			Plasmapheresis (*γ*)			*p* value
		Survival	Death		Survival	Death	Subtotal	Survival	Death	Subtotal	Survival	Death	Subtotal	
Year	2000	24	2	26	20	2	22	0	0	0	4	0	4	0.71
	2001	13	2	15	12	2	14	0	0	0	1	0	1	0.87
	2002	18	2	20	17	2	19	0	0	0	1	0	1	0.90
	2003	19	4	23	13	3	16	2	1	3	4	0	4	0.50
	2004	29	0	29	26	0	26	1	0	1	2	0	2	ND
	2005	27	0	27	23	0	23	2	0	2	2	0	2	ND
	2006	18	0	18	17	0	17	0	0	0	1	0	1	ND
	2007	19	2	21	15	2	17	0	0	0	4	0	4	0.65
	2008	21	0	21	15	0	15	0	0	0	6	0	6	ND
	2009	29	2	31	17	0	17	7	0	7	5	2	7	0.03^*∗*^
	2010	21	2	23	18	0	18	1	0	1	2	2	4	<0.01^*∗*^
	2011	21	2	23	15	1	16	2	0	2	4	1	5	0.57
	2012	27	2	29	23	1	24	2	0	2	2	1	3	0.16
	2013	26	1	27	23	1	24	2	0	2	1	0	1	0.94
	2014	40	1	41	33	0	33	1	0	1	6	1	7	0.08

Total		352	22	374	287	14	301	20	1	21	45	7	52	0.04^*∗*^

*∗* indicates *p* < 0.05; the comparison is significant between *α* and *γ*, as well as between *α*, *β*, and *γ*; ND indicates that death is a constant, which is zero.

**Table 3:** Comparison of laboratory data according to treatment modality of systematic lupus erythematosus patients.

Characteristics *n* = 374	Plasma exchange (*α*)		Combination (*β*)		Plasmapheresis (*γ*)		*p* value	Significance
	*n* = 301		*n* = 21		*n* = 52			
	Mean	SD	Mean	SD	Mean	SD		
Age	38.92	16.18	30.14	13.30	34.25	12.44	0.01^*∗*^	*αβ*
Leukocyte	6.63	2.74	5.41	2.86	6.70	2.71	0.52	
Neutrophil (%)	69.85	13.27	60.80	18.19	75.25	10.12	0.20	
Lymphocyte (%)	22.17	11.93	27.60	17.23	18.23	9.37	0.44	
Monocyte (%)	6.14	2.77	7.38	2.66	5.63	2.32	0.55	
Platelet	201.22	79.40	191.71	92.42	204.85	75.62	0.94	
Hemoglobin	11.25	2.12	12.09	.96	12.84	1.53	0.02^*∗*^	*αγ*
Hematocrit	34.06	6.12	36.67	2.45	38.01	4.43	0.02^*∗*^	*αγ*
C-reactive protein	15.12	28.74	1.83	.83	4.70	5.52	0.65	
Erythrocyte sediment rate	40.06	31.10	34.00	x	13.80	17.63	0.20	
Creatinine	0.92	0.95	0.61	0.02	0.61	0.06	0.66	
High-density lipoprotein	62.00	18.54	72.00	x	40.50	0.71	0.25	
Low-density lipoprotein	104.85	37.99	77.00	x	79.00	45.25	0.55	
Rheumatoid factor	14.37	4.97	x	x	11.50	x	0.67	
Anti-nuclear antibody (times of dilution)	250.00	429.49	x	x	30.00	38.30	0.32	
Beta 2-glycoprotein I	10.77	22.78	6.05	3.89	21.33	33.59	0.44	
Anti-cardiolipin IgG	12.45	18.92	6.33	2.81	37.63	44.60	0.11	
Anti-cardiolipin IgM	4.57	8.56	1.93	1.53	3.60	4.25	0.85	
Anti-Ro IgG	138.54	112.08	148.95	115.20	141.41	133.95	0.98	
Anti-La IgG	53.12	110.51	54.48	130.08	66.33	138.36	0.94	
Anti-U1 ribonuclear protein IgG	30.53	69.08	86.17	131.53	48.43	95.04	0.24	
Anti-Smith IgG	6.31	20.23	20.52	48.74	14.45	36.45	0.33	
Anti-Scl70 IgG	0.43	0.39	0.35	.35	0.35	0.31	0.83	
Anti-Jo1 IgG	0.34	0.28	0.40	x	0.25	0.30	0.83	
Hepatitis B surface antigen titer	1132.40	2041.86	x	x	0.00	x	0.61	

SD: standard deviation. *∗* indicates *p* < 0.05.

**Table 4:** Subgroup analysis of comorbidities, treatment methodology including plasma exchange, or plasmapheresis between survivors and mortalities among 374 systemic lupus erythematosus patients.

*N* = 374	Survivors	Mortalities	*p* value	Multivariate analysis	95% confidence interval
	352	22		*p* value	
Female	308	21	0.23		
Comorbidities:					
Rheumatoid arthritis	5	0	0.74		
Systemic sclerosis	1	0	0.94		
Sicca syndrome	7	0	0.65		
Dermatomyositis	1	0	0.94		
Polymyositis	1	0	0.94		
Diabetes mellitus	5	2	0.06		
Hepatitis, non-B, non-C	7	0	0.65		
Gouty arthritis	1	0	0.94		
Rheumatism	3	0	0.83		
Hyperlipidemia	4	0	0.78		
Hypertension	31	2	0.60		
Bronchitis	1	0	0.94		
Fibrositis	3	0	0.83		
Cytomegalovirus infection	2	0	0.89		
Chronic hepatitis B infection	1	0	0.94		
Major depression	1	0	0.94		
Chronic hepatitis C infection	1	0	0.94		
Peripheral neuropathy	2	0	0.89		
Therapy and examination:					
Bone marrow biopsy	17	0	0.35		
Kidney biopsy	21	0	0.27		

Treatment methodology					
Plasma exchange (PE)	309	15	0.02^*∗*^	0.02^*∗*^	0.12–0.81
Plasmapheresis (PP)	56	7	0.04^*∗*^	x	1

Death (PP versus PE)	15 vs. 7		0.04^*∗*^	0.02^*∗*^	3.21 (1.24–8.34)

*∗* indicates *p* < 0.05.

## Data Availability

All the underlying research materials related to our article can be accessed on demand by email notification.

## Supplementary Materials

Supplementary Materials Supplementary Table 1 shows the equipment of plasmapheresis (PP) and the plasma exchange (PE) services used in Kaohsiung Chang Gung Memorial Hospital.
