# Supplementary material for: Lower In-Hospital Mortality with Plasma Exchange than Plasmapheresis in a Subgroup Analysis of 374 Lupus Patients
Source: Biomed Res Int. 2018 May 2;2018:9707932. doi: 10.1155/2018/9707932 (PMC5954964; doi:10.1155/2018/9707932)
Supplement: Supplementary Materials — Supplementary Table 1 shows the equipment of plasmapheresis (PP) and the plasma exchange (PE) services used in Kaohsiung Chang Gung Memorial Hospital. [file 9707932.f1.docx]

Supplement Table 1. The plasmapheresis (PP) and the plasma exchange (PE) services in Kaohsiung Chang Gung Memorial Hospital.

|  | Plasmapheresis (PP) | Plasma exchange (PE) |
| --- | --- | --- |
| Machine | HF-440 | HF-440 |
| Hollow fiber | Plasmacure | Plasmacure |
| Fractionator | Evaflux 4A | no fractionator |
| Plasma infusion | Fractionized plasma from patient | Fresh frozen plasma from others |
